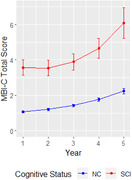# Five‐year Trajectory of Mild Behavioral Impairment in People with Subjective Cognitive Complaints

**DOI:** 10.1002/alz.087995

**Published:** 2025-01-03

**Authors:** Byron Creese

**Affiliations:** ^1^ Brunel University London, London United Kingdom

## Abstract

**Background:**

Mild Behavioral Impairment (MBI) describes a spectrum of late‐life onset, sustained neuropsychiatric symptoms grouped in five domains: mood/anxiety, apathy, impulse dyscontrol, social inappropriateness and psychosis. MBI is a well‐established correlated of incident dementia and is associated with PET amyloid and blood biomarker profiles consistent with early AD however little is known about the trajectory of MBI. This study set out to determine the course of MBI symptoms in a cohort of older adults with and without subjective cognitive complaints (SCC).

**Method:**

Data from 3,428 adults aged over 50 were analysed. All had five years of annual follow up assessments. SCC was determined by self‐completed IQCODE scale at baseline(mean score <3.3: normal cognition (NC); >3.3: SCC). MBI was assessed using the MBI Checklist (MBI‐C) (higher scores indicating more severe symptoms). Change in MBI‐C score by SCC status was conducted using zero‐inflated negative binomial models. A random intercept was included to allow for correlations between repeated measurements on the same individual. Trajectories of mood/anxiety and apathy were also analysed. The other three domains were not due to low counts of non‐zero scores.

**Result:**

The mean age at baseline was 64 and 78% of the sample were women. Fifteen percent (n = 514) had SCC. The SCC group had higher MBI scores than NC. For the SCC group, there was a statistically significant exponential increase in MBI‐C score over 5‐years. At baseline, total MBI‐C score was 3.5[3.1‐4], rising to 4.6[4.1‐5.2] in year 4 and 6[5.2‐7] in year 5 (Figure 1). For NC, the increase over 5 years was more modest, rising from 1 at baseline to 2.2 by year 5.

**Conclusion:**

To our knowledge this is largest study of trajectories of MBI symptoms. We show that symptoms are not stable but show an exponential increase over time in those with SCC, while in those with normal cognition the overall trend was flat. These are population averaged estimates and there will be a degree of variability in the data which may warrant more individual‐level prediction modelling in future. As well as being a marker of cognitive risk, SCC may also represent a risk state for worsening neuropsychiatric symptoms.